# Graphene Nanoflake- and Carbon Nanotube-Supported Iron–Potassium 3D-Catalysts for Hydrocarbon Synthesis from Syngas

**DOI:** 10.3390/nano12244491

**Published:** 2022-12-19

**Authors:** Sergei A. Chernyak, Dmitrii N. Stolbov, Konstantin I. Maslakov, Ruslan V. Kazantsev, Oleg L. Eliseev, Dmitry O. Moskovskikh, Serguei V. Savilov

**Affiliations:** 1 Department of Chemistry, Lomonosov Moscow State University, Leninskie Gory 1-3, Moscow 119991, Russia; 2 N.D. Zelinsky Institute of Organic Chemistry, Russian Academy of Sciences, Leninsky prosp. 47, Moscow 119991, Russia; 3 Research Center Structural Ceramic Nanomaterials, National University of Science and Technology, “MISIS”, Moscow 119049, Russia

**Keywords:** syngas conversion, Fischer–Tropsch synthesis, graphene nanoflakes, carbon nanotubes, N-doping, potassium promotion, carbon-encapsulated nanoparticles, iron carbide

## Abstract

Transformation of carbon oxides into valuable feedstocks is an important challenge nowadays. Carbon oxide hydrogenation to hydrocarbons over iron-based catalysts is one of the possible ways for this transformation to occur. Carbon supports effectively increase the dispersion of such catalysts but possess a very low bulk density, and their powders can be toxic. In this study, spark plasma sintering was used to synthesize new bulk and dense potassium promoted iron-based catalysts, supported on N-doped carbon nanomaterials, for hydrocarbon synthesis from syngas. The sintered catalysts showed high activity of up to 223 μmol_CO_/g_Fe_/s at 300–340 °C and a selectivity to C_5+_ fraction of ~70% with a high portion of olefins. The promising catalyst performance was ascribed to the high dispersity of iron carbide particles, potassium promotion of iron carbide formation and stabilization of the active sites with nitrogen-based functionalities. As a result, a bulk N-doped carbon-supported iron catalyst with 3D structure was prepared, for the first time, by a fast method, and demonstrated high activity and selectivity in hydrocarbon synthesis. The proposed technique can be used to produce well-shaped carbon-supported catalysts for syngas conversion.

## 1. Introduction

Rational utilization of CO_2_ is one of the most important challenges nowadays, due to global warming and irreversible climate changes. One of the possible routes for reducing the amount of CO_2_ is its transformation into valuable chemicals, such as hydrocarbons. This can be realized using Fischer–Tropsch synthesis (FTS), in which CO_2_ is initially transformed to CO via the reversible water gas shift process, and, then, CO is hydrogenated towards different hydrocarbons [1]. FTS is an industrial route to produce synthetic fuels, wax, light olefins, and lubricants from a diversity of feedstocks, such as natural gas, coal, and biomass [2,3,4]. Two main types of catalysts are used in this process: iron- and cobalt-based ones. The CO:H_2_ ratio determines the choice of the catalyst type. The cobalt catalysts are commonly used for gaseous raw materials with a CO:H_2_ ratio of 1:2. The iron catalysts are more suitable for biomass and coal transformation, due to their high flexibility to syngas composition [5]. At the same time, the iron catalysts are usually promoted with alkali and alkaline–earth metals, which increases their selectivity and stability [6,7]. Potassium is the most popular promoter as it enhances the formation of iron carbides and increases the selectivity to C_2+_ hydrocarbons [8].

Carbon nanomaterials have been intensively studied in the past decade as supports for both iron and cobalt FTS catalysts, and their strong effects on catalyst performance demonstrated [9,10,11,12]. These supports are chemically inert and provide better metal dispersion than oxide materials, which increases the total number of active surface sites and enhances the catalyst performance. At the same time, several drawbacks prevent the large-scale application of carbon supports. Among them, the low bulk density and “powderiness” are the most crucial ones. In this regard, compaction and activation of FTS catalysts by spark plasma sintering (SPS) was found to be an effective approach to overcome these drawbacks [13]. This technique implies the simultaneous action of high pressure and pulsed direct current that induces a fast heating of the sample at a speed of ~100 degrees per minute to temperatures as high as 2000 °C. As a result of the high sintering speed, SPS preserves the pore structure of the catalyst and allows formation of active meta-stable species in the energy saving process [14,15,16]. When applied to a metal oxide–carbon system, it also leads to the formation of an unique 3D nanostructured carbon framework, in which reduced carbon-encapsulated metal nanoparticles are embedded [17]. Carbon shells stabilize metal crystallites preventing their oxidation in air and sintering during FTS.

Carbon-encapsulated iron and cobalt nanoparticles are known to be effective in FTS since carbon shells do not block the active sites, but strongly enhance the stability of the catalysts towards sintering [18,19]. Pyrolytic approaches are mainly used for the synthesis of such types of encapsulated particles. They include pyrolysis of an iron salt with organic compounds or carbon materials, pyrolysis of iron-containing organic compounds, CVD and hydrothermal preparation [20,21,22,23]. The iron carbide formation during pyrolysis of Prussian blue was studied in [21], and the authors showed that the type of carbide and, hence, the catalyst performance in FTS depended on the preparation temperature. Carbon-encapsulation of the catalyst particles facilitates magnetic excitation of the catalyst and promotes its performance in different catalytic processes [24].

Potassium is known to improve the selectivity of FTS catalysts [25,26], while N-doping of nanocarbon materials is a powerful approach for tuning their electronic properties and defectiveness, increasing metal dispersion and enhancing reduction of supported metal oxides [27,28,29,30,31]. Since the surface of pristine carbon nanomaterials is nonpolar it is usually functionalized with oxygen-containing groups, that anchor metal particles, thereby increasing the dispersion of the active component. Nitrogen species may be an alternative to oxygen functionalities, and their incorporation into carbon support allows the oxidation step during catalyst preparation to be avoided [27].

This work studied the formation of the 3D structure of carbon-supported iron catalysts under SPS. In this structure, the support framework is formed from N-doped graphene nanoflakes (N-GNFs) or N-doped carbon nanotubes (N-CNTs). We demonstrate that SPS is an effective approach for the synthesis of compact, active and selective N-doped carbon-supported catalysts for FTS. The N-GNF-supported catalyst is more active than the N-CNT-supported one, which is attributed to the higher content of N atoms in the carbon support and higher dispersion of iron in the former catalyst.

## 2. Experimental

The N-GNFs and N-CNTs were synthesized at 800 and 750 °C, respectively, by pyrolysis of acetonitrile over a MgO template (for N-GNFs), or Co-Mo/MgO catalyst (for N-CNTs), in a quartz horizontal tubular reactor, described in detail in [32,33,34]. In brief, the MgO template was prepared by precipitation of magnesium oxalate from magnesium nitrate by ammonia oxalate and further calcination of the dried powder at 500 °C. The Co-Mo/MgO catalyst was prepared by the combustion approach, in which water solutions of Co and Mg nitrates, ammonia molybdate and citric acid were mixed and heated initially to 250 °C and, then, to 500 °C. The synthesized template (catalyst) was placed inside the reactor. The reactor was heated up to the desired temperature in a nitrogen flow and then the flow was switched to bubble through the acetonitrile and passed over the template for 30 min for N-GNFs, and for 5 h for N-CNTs. Then, the reactor was cooled down to room temperature and the produced material was refluxed with ~19% HCl water solution for 4 h to remove the template (catalyst). After that, the materials were filtered, washed with distilled water, and dried overnight at 110 °C.

The catalyst precursor for SPS was prepared by impregnation of the obtained N-doped materials with iron nitrate and potassium carbonate water solution, followed by evaporation of the solvent in a rotary evaporator, with constant stirring, similar to that used for the Co catalyst synthesized in [27]. The calculated concentrations of iron and potassium were 10 and 0.5 wt.%, respectively. To decompose the nitrate the produced materials were calcined in a vertical tubular quartz reactor in N_2_ flow at 300 °C for 2 h. The synthesized catalysts were designated as “FeKNGNF” and “FeKNCNT”.

The SPS was carried out in a Labox-625 system (Sinterland, Japan). The FeKNGNF or FeKNCNT powder was placed in a graphitic die, heated in a vacuum of 10^−2^ Torr by pulsed direct current up to 700 °C (heating rate of 100 °C/min), while simultaneously applying a pressure of 30 MPa, then sintered for 5 min and cooled down to room temperature. The sintered catalysts were named as “FeKNGNF700” and “FeKNCNT800”. The sintering temperature of 700 °C was chosen based on previously published results for GNF-supported iron catalysts. Mainly, the iron oxide phase was detected in the Fe/GNFs catalyst, sintered at 600 °C, while sintering at 800 °C produced predominantly huge carbide agglomerates [35]. The sintering temperature of 800 °C was chosen for the N-CNTs-supported catalyst according to the data for undoped CNTs [13].

Catalysts were tested in a down flow fixed-bed stainless steel reactor with an ID of 16 mm. The catalyst (270 mg) was diluted with 2 cc of quartz sand and placed in the isothermal zone of the reactor. The synthesis gas (CO:H_2_ molar ratio of 1:1 plus 5% Ar as an internal standard) was passed through the reactor with a flow rate of 5 NLg_cat_^−1^ h^−1^ at a pressure of 20 bar. The temperature was slowly increased up to 300 °C. For the N-GNF-supported catalyst the temperature was further raised to 340 °C and, then, reduced back to 300 °C. The composition of gaseous products (C_1_–C_4_ hydrocarbons and CO_2_) was determined on a Kristallyuks-4000M gas chromatograph (Meta-Khrom, Yoshkar-Ola, Russia) equipped with two packed steel columns (1.5 m × 2.1 mm) filled with HayeSep phase (60% divinylbenzene and 40% ethylene glycol dimethacrylate) and NaX molecular sieves. Helium, as a carrier gas, and a TCD detector were used. Liquid hydrocarbons were collected in a container at ambient temperature and analyzed by an Avtokhrom-3700 instrument (Alltech^TM^ quartz capillary column of 30 m × 0.25 mm, SE 30 stationary phase, nitrogen as a carrier gas, PID).

The CO conversion was calculated as follows:

$$X_{CO}=\frac{moles\;CO\;converted}{moles\;of\;CO\;passed}\times 100\%$$

Selectivity to each gaseous product was calculated by the formula:

$$S=\frac{moles\;CO\;converted\;to\;the\;product}{moles\;of\;converted\;CO}\times 100\%$$

The selectivity to C_5+_ hydrocarbons was calculated based on a total mass balance of carbon in the feed and gaseous products:

$$S=100\%-S_{CO2}-S_{CH4}-S_{C2-C4}$$

The activity of the catalyst was calculated as the iron time yield (FTY) in micromoles of converted CO per gram of iron per second.

The catalysts were analyzed by XPS, XRD, and TEM. XPS spectra were acquired on an Axis Ultra DLD spectrometer (Kratos Analytical, Manchester, UK) with a monochromatic Al Kα source. Pass energies of 160 and 40 eV were used, respectively, for survey spectra and high-resolution scans. A JEM 2100 F/Cs microscope (JEOL Ltd., Tokyo, Japan), operated at 200 kV, was used for the TEM study. The sample was dispersed in methanol, dropped on a polymer-covered copper grid and dried. A Stoe Stadi P diffractometer (Darmstadt, Germany) with a Ni-filtered CuKα X-ray tube was used for XRD measurements.

## 3. Results and Discussion

### 3.1. Catalyst Structure

According to the XPS data, the synthesized N-GNFs contained ~9 at.% of nitrogen, mainly as pyridine, pyrrole, and quaternary/graphitic species [27,34]. The same nitrogen functionalities were detected in N-CNTs, but at much lower concentrations of only 1–3 at.% in total [33].

**Figure 1:**
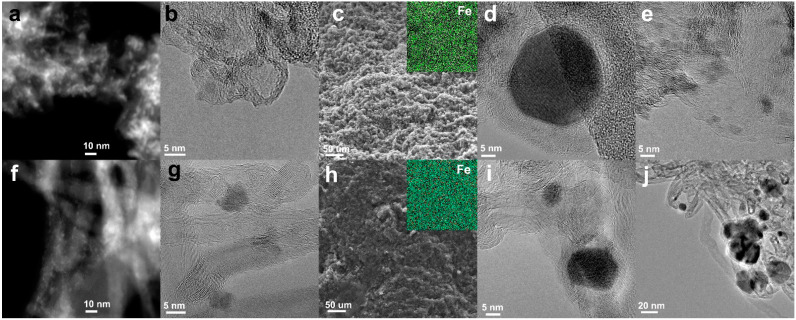
TEM images of FeKNGNF (**a**,**b**), sintered FeKNGNF700 (**d**,**e**), FeKNCNT (**f**,**g**) and FeKNCNT800 (**i**,**j**); SEM images of FeKNGNF700 (**c**) and FeKNCNT800 (**h**) (insets show corresponding EDX iron mappings).

The support of unsintered FeKNGNF consisted of uniformly distributed stacked graphene sheets of 5–30 nm in size with bent edges (Figure 1a,b). Iron oxide nanoparticles of ~2–5 nm in diameter were well-distributed over the support. Such a low particle size was explained by the large number of anchoring sites, such as edge carbon atoms and nitrogen functionalities, within the graphene layers of N-GNFs. In contrast to the unsintered sample, FeKNGNF700, along with many tiny particles of 2–5 nm in size, contained a certain number of huge crystallites (Figure 1d,e). The phase analysis of these crystallites via Fourier transformation of HRTEM images revealed the Fe_3_C iron carbide (cementite) phase, while smaller particles seemed to contain iron oxide. Thus, the sintered FeKNGNF700 sample contained both nanosized iron oxide particles and large iron carbide crystallites. Nanotubes in the unsintered FeKNCNT showed a lot of defects and partitions inside the channels, which was typical for N-doped nanotubes [33]. Iron oxide nanoparticles were uniformly distributed over the sample surface (Figure 1f,g). The SPS of this material led to the formation of 10–50 nm *bcc*-Fe crystallites covered with graphitic shells (Figure 1i,j). The SEM images showed the grain morphology of the sintered samples (Figure 1c,h), which is typical for SPS-treated materials [36]. The EDX elemental mappings demonstrated the uniform macro-level distribution of iron after sintering.

**Figure 2:**
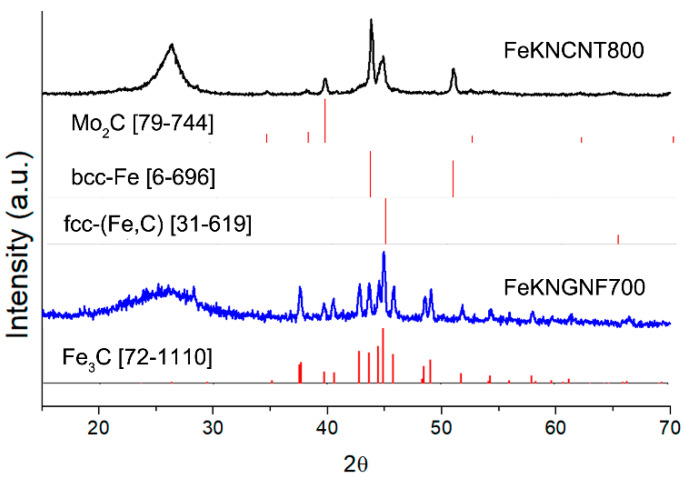
XRD pattern of fresh FeKNGNF700 and FeKNCNT800 catalysts.

The XRD pattern of FeKNGNF700 (Figure 2) showed only the reflections of the cementite (Fe_3_C) phase. Iron oxide phases were not detected in this sample by XRD, because of the low size of oxide particles, as was demonstrated by TEM. According to the XRD data, FeKNCNT800 contained a mixture of the *bcc*-Fe and austenite (*fcc*-Fe,C) phases and the residual Mo_2_C phase from the growth catalyst. The different reducibility of iron oxide particles in the N-GNF- and N-CNT-supported samples under SPS resulted from the different electrical conductivities of GNFs and CNTs. The high aspect ratio of CNTs enhanced the electrical and thermal conductivities of the CNT-supported material, which more uniformly distributed Joule heat during SPS. In contrast to CNTs, a large number of inter-particle contacts reduced the overall conductivity of the GNF-based material. This led to nonuniform heat distribution during SPS, which, in its turn, resulted in the presence of both iron carbide and oxide particles.

The calculated crystallite size of the cementite phase in FeKNGNF700 was ~40 nm, while those of the *bcc*-Fe and *fcc-*(Fe, C) phases in FeKNCNT800 were ~30 and 15 nm, respectively, which correlated with the large crystallite size determined by TEM.

### 3.2. Catalytic Performance

The sintered catalysts were tested in FTS, initially at 300 °C. The catalytic performances of the samples are summarized in Table 1. The FeKNCNT800 showed almost two times higher CO conversion than the N-GNF supported sample. At the same time, higher activity in the water–gas shift (WGS) reaction and higher methane selectivity over FeKNCNT800 led to the almost similar C_2+_ yields of the two catalysts. Higher activity of FeKNCNT800 could be assigned to the higher concentration of carbon-protected Fe-containing particles in the catalyst and their smaller average size. It should also be noted that this catalyst contained a noticeable amount of the Mo_2_C phase that itself could be active both in WGS and FTS [25]. At the same time, pristine nanotubes with a Mo admixture were inactive in FTS because Mo is a residue of the growth catalyst and, hence, it is strongly encapsulated inside nanotube channels.

**Figure 3:**
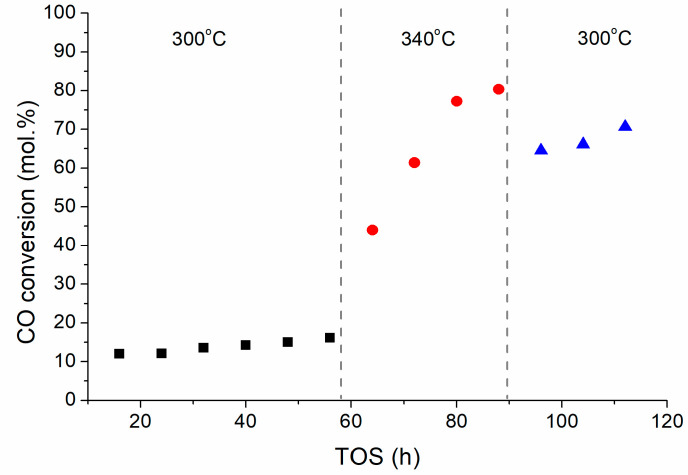
CO conversion over FeKNGNF700 versus TOS at different temperatures (20 bar, 5 NL/g_cat_/h, CO:H_2_ = 1:1).

Iron carbide is known to be the active phase in iron-containing FTS catalysts. As the FeKNGNF700 contained a large number of ultra-small oxide particles, we decided to additionally test this catalyst at a higher temperature to check the in-situ carbidization of the oxide nanocrystals and to enhance catalyst performance. For this purpose, when the test at 300 °C (first stage) was completed, the temperature was increased to 340 °C (second stage) and, after reaching a quasi-steady state the reactor was cooled down again to 300 °C (third stage) (Figure 3).

The CO conversion in the first stage was rather low and slowly increased with TOS from ~12 to ~16%. After increasing the temperature up to 340 °C, the CO conversion sharply grew, up to ~80% in 32 h. This fact could be explained by the in-situ carbidization of ultra-small iron oxide nanoparticles in syngas. The subsequent cooling to 300°C did not reduce conversion below ~70% because the catalyst was totally carbidized. Along with conversion, the activity of the catalyst in the WGS reaction also increased, inducing selectivity to CO_2_ (about 30%) (Table 2). This behavior was typical when partial water pressure in a system grows with increase in conversion [37,38]. The selectivity to hydrocarbon fractions remained similar at three stages of the test: the C_5+_ fraction dominated (65–70%), while the portions of C_2_–C_4_ and C_5+_ olefins were 55–60 and 30–40%, respectively. Moderate chain growth coefficients (α) of ~0.75–0.78 and ~0.67–0.70 were observed, respectively, for liquid paraffins and olefins. These values were typical for Fe-based catalysts and higher than those observed for Co-based catalysts (about 0.9).

It is worth noting that high portions of olefins were detected for the FeKNGNF700 and FeKNCNT800 catalysts, both in the gas and liquid fractions. These products are highly desirable, as they are widely used as intermediates in the synthesis of polymers, surfactants and lubricants [39].

Table 3 compares the catalytic performances of the FeKNGNF700 and FeKNCNT800 catalysts with the literature data for similar sintered and unsintered unpromoted iron catalysts. While the C_5+_ selectivity of the GNF-supported sintered catalysts (FeKNGNF700 and FeGNF800) was almost the same (~70 mol.%), the activity of the K-promoted catalyst synthesized in this work was two-times higher than that of the sintered unpromoted catalyst. There may be several reasons for this fact. Doping of carbon support with nitrogen noticeably increases the metal–support interaction, due to both the electron density redistribution and appearance of surface defects. For example, generation of graphitic and quaternary nitrogen functionalities implies the incorporation of electron reach dopants into graphene layers, which increases the electron density in the material. These changes enhance the interaction between metal ions and the surface during the catalyst synthesis stage. At the same time, pyridine and pyrrole fragments introduce vacancies in graphene layers forming new sites for the nanoparticle stabilization. Earlier, the effect of nitrogen doping on the dispersity and stability of catalysts supported on GNFs and other graphene-based nanostructured materials was demonstrated [27,29,31]. Another important factor of nitrogen doping that affects the catalyst performance in FTS is that the addition of electrons may enhance CO activation and dissociation. For example, N-groups, especially pyrroles, play an essential role in CO adsorption and dissociation, enhancing the activity, selectivity, and stability of the catalysts supported on N-CNTs [40,41].

Promotion of the catalyst with potassium could also be a reason for enhancement in its catalytic performance. Similar to nitrogen doping, potassium increases the electron density in the catalyst, enhancing its ability to dissociate CO and intensifying the iron carbide formation [8]. Since iron carbide is a well-known active site for FTS, this promotion positively affects catalyst performance. The CO molecules penetrate to the carbon-encapsulated particles through defects in the carbon shells and dissociate on the particle surface enriched with electrons by potassium species. Chain growth proceeds via the carbide mechanism by interaction of C_x_H_y_ surface species with hydrogenated surface carbon atoms [44]. Table 3 also shows higher activity of the FeKNGNF700 catalyst than those of similar unsintered catalysts tested under the same conditions. The SPS can affect the catalyst structure in the following ways. Firstly, densification of the material leads to more intimate contact between the iron-based particles and the carbon support, which enhances carbide formation and electron transfer between nanoparticles. Moreover, a catalyst prepared by SPS already contains a noticeable amount of carbide phase and shows FTS activity even without pre-reduction. This activity strongly increases during a high-temperature FTS test.

Opposite to N-GNFs, N-CNTs synthesized from acetonitrile contained a small amount of nitrogen, only up to 3 at.%. This amount may be insufficient to strongly affect the metal–support interactions and enhance CO adsorption during FTS. Lower activity of FeKNCNT800, compared to the undoped CNT-supported iron catalyst, may also be explained by the large particle size in the former catalyst, which also results from lower metal–support interaction and insufficient metal particle stabilization during SPS.

### 3.3. Study of Spent Catalyst

**Figure 4:**
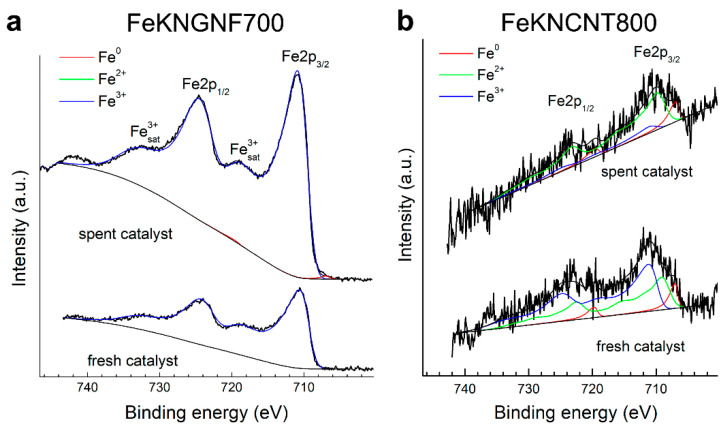
The Fe 2p XPS spectra of fresh and spent catalysts: FeKNGNF700 (**a**) and FeKNCNT800 (**b**).

The spent catalysts were analyzed by XPS and XRD. According to the XPS data, the surface iron content in FeKNGNF700 strongly decreased after the catalytic test, which pointed to the formation of surface carbides and partial carburization of iron particles (Table 4). As XPS is a surface-sensitive technique, with an analysis depth of only a few nanometers, the formation of surface carbide and carbon layers reduced the iron concentration on the surface (according to the XRF data, the iron concentration in the fresh catalyst was 12.6 wt.%, while that of potassium was 0.6 wt.%). Only Fe^3+^ species were detected in the Fe 2p spectrum of the fresh catalysts (Figure 4a), which confirmed the XRD data on the predominantly oxidized state of iron in this sample. The spectrum of the spent catalyst, along with Fe^3+^ species, showed a small contribution (~3%) from non-oxidized (metallic or carbide) iron. During FTS, iron carbides were formed in the catalysts, butmagnetite formation was possible on the surface during CO dissociation. Air exposure of the sample after the test could also oxidize iron on the surface. A noticeable decrease in the O and N concentrations during FTS was detected, which might reflect, firstly, the coverage of the surface with amorphous carbon and, secondly, partial thermal defunctionalization of GNFs [34,45].

A very small amount of iron was observed, both in the fresh and spent N-CNT-supported catalyst (Table 4), which pointed to coverage of the iron particles with thick graphitic shells. The Fe 2p spectra of FeKNCNT800 (Figure 4b) were noisy, because of the low iron concentration on the surface. Nevertheless, they showed the contribution of both metallic and oxidized iron species.

**Figure 5:**
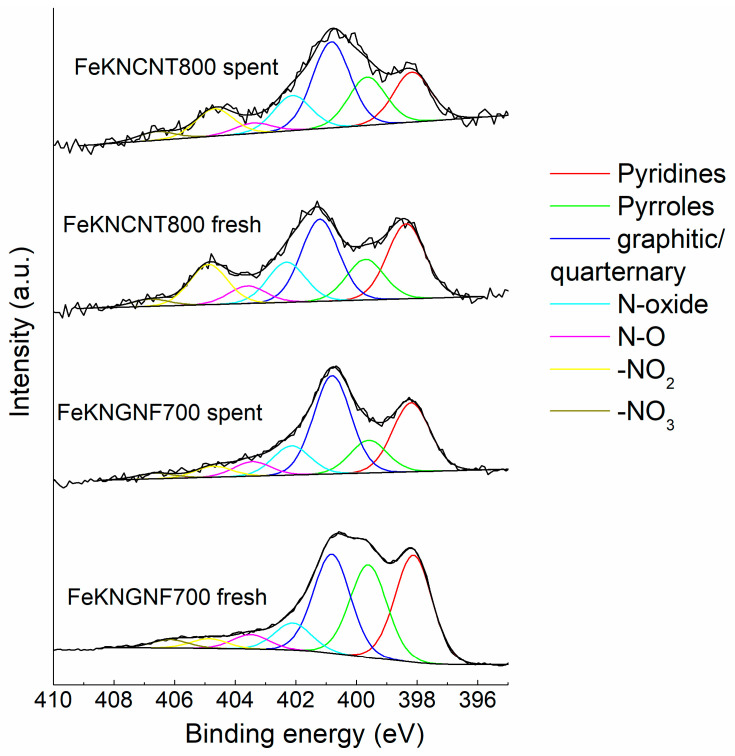
The N1s XPS spectra of fresh and spent catalysts. Components in the spectra were assigned according to Refs. [46,47,48,49].

Figure 5 demonstrates the changes in the N1s spectra of the catalysts after catalytic tests. Nitrogen functionalities were mostly stable in N-CNTs, and only a slight decrease in the contents of pyridines and −NO_2_ fragments was detected. In contrast, the amount of pyrrolic and pyridinic groups in FeKNGNF700 strongly decreased after the test. Since pyridines and pyrroles are associated with local defects (holes) in graphene sheets, they are probably the anchoring sites for iron particles. During FTS, especially at a relatively high temperature of 340 °C these groups can decompose under etching of the carbon surface with hydrogen, while more stable quaternary or graphitic nitrogen species remain on the support surface [27,34].

**Figure 6:**
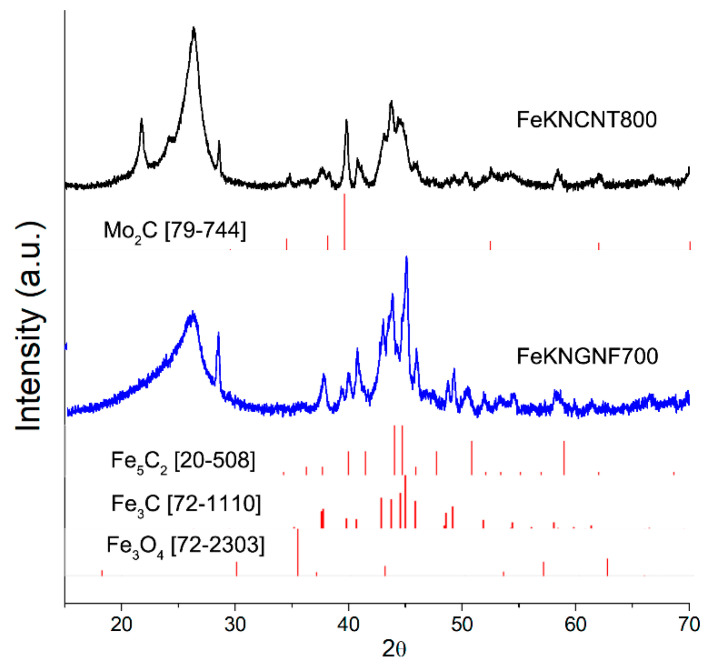
XRD pattern of spent FeKNGNF700 catalyst.

The XRD analysis of both the spent catalysts revealed the formation of Hägg carbide during FTS (Figure 6). This phase transformation explained the growth of FeKNGNF700 activity during the FTS test at an increased temperature of 340 °C. The Hägg carbide sites were more active in FTS than the cementite ones, and the promotion of the catalyst with alkaline metals enhanced the carbidization ability [8,50]. A weak XRD peak of iron oxide phase at 35.6° could be attributed to a thin oxide layer around the carbide particles. This fact correlated with the XPS data on the mostly oxidized state of iron species on the surface of the spent catalyst. Narrow peaks at ~21.8, 24.3 and 28.6° in the XRD patterns of the spent catalysts corresponded to admixture of silica particles, used as barrier layers inside the reactor.

## 4. Conclusions

Spark plasma sintering was applied to synthesize bulk carbon-based catalysts for syngas conversion towards hydrocarbons. Potassium-promoted iron catalysts, supported on N-doped graphene nanoflakes and N-doped carbon nanotubes, were produced by this technique, which enhanced the activity of the GNF-based catalyst in FTS up to 196 μmol_CO_/g_Fe_/s, preserving a high C_5+_ selectivity of about 70 mol.% with a noticeable portion of olefins (up to ~40%). The sintering effect was not so strong for the N-CNT supported catalyst, and its performance was lower than that of the N-GNF-supported catalyst, due to the higher particle size and lower nitrogen content in the N-CNT support. The positive effect of N-doping on the GNF support was ascribed to increase in both the metal–support interaction and surface defectiveness, which enhanced iron nanoparticle anchoring and their stabilization.

## Figures and Tables

**Table 1:** Conversion and selectivity data for FeKNGNF700 and FeKNCNT800 catalysts in FTS (20 bar, 300 °C, 5 NL/g_cat_/h, CO:H_2_ = 1:1).

Catalyst	X_CO_ (mol.%)	S_CO2_ (mol.%)	Selectivity to HC Fractions (mol.%)				Olefins in C_2_–C_4_ (mol. %)	Liquid Fraction			C_2+_ Yield (mol.%)
			CH_4_	C_2_–C_4_^=^	C_2_–C_4_^−^	C_5+_		α_n-par_	α_n-olef_	Olefins (mol. %)	
FeKNGNF700	16.1	7.8	10.4	11.7	7.3	70.6	62	n/d	n/d	n/d	13.3
FeKNCNT800	28.1	29.7	14.9	10.8	9.8	64.4	52	0.865	0.783	30.2	16.8

**Table 2:** Performance of FeKNGNF700 catalyst in FTS (20 bar, 5 NL/g_cat_/h, CO:H_2_ = 1:1) at different temperatures.

T (°C)	X_CO_ (mol.%)	S_CO2_ (mol.%)	Selectivity to HC Fractions (mol.%)				Olefins in C_2_–C_4_ (mol.%)	Liquid Fraction		
			CH_4_	C_2_–C_4_^=^	C_2_–C_4_^−^	C_5+_		α_n-par_	α_n-olef_	Olefins (mol.%)
300 (initial)	16.1	7.8	10.4	11.7	7.3	70.6	62	n/d	n/d	n/d
340	80.3	29.3	12.2	12.0	9.5	66.3	56	0.781	0.695	31.6
300 (after 340)	70.6	30.1	9.6	12.4	7.9	70.1	61	0.746	0.666	38.9

**Table 3:** Performances of FeKNGNF700 and FeKNCNT800 catalysts in FTS compared with the literature data for unpromoted carbon supported Fe-based catalysts.

Catalyst	Test Conditions	CO-Free C_5+_ Selectivity (mol.%)	FTY (μmolCO/gFe/s)	Ref.
FeKNGNF700	300 °C, 20 bar, 5 NL/g/h	70.1	196	This work
FeGNF800		73.6	72	[35]
FeGNF reduced		68.3	102	[35]
FeKNCNT800		64.4	78	This work
FeCNT800		77.6	203	[13]
Fe_5_C_2_@C	320 °C, 15 bar, 8 NL/g/h	52.7	150	[42]
Fe/N-CNTs	275 °C, 8 bar, 2400 h^−1^	60.9	55	[41]
10%Fe/AG(12h)-W	320 °C, 20 bar, 4.5 NL/g/h	40	185	[31]
Fe/G-C	340 °C, 20 bar, 8 NL/g/h	70.9	293	[43]

**Table 4:** The XPS surface compositions (at.%) of fresh and spent FeKNGNF700 catalysts.

Catalyst		Fe	K	C	N	O
FeKNGNF700	Fresh	4.0	0.3	84.7	3.3	7.7
	Spent	0.9	n/d	95.1	1.0	3.0
FeKNCNT800	Fresh	0.1	0.2	97.8	0.8	1.1
	Spent	<0.1	0.1	97.9	0.6	1.4

## Data Availability

The data presented in this study are available on request from the corresponding author.
